# Human Xylosyltransferase I—An Important Linker between Acute Senescence and Fibrogenesis

**DOI:** 10.3390/biomedicines11020460

**Published:** 2023-02-04

**Authors:** Vanessa Schmidt, Justus Ohmes, Thanh-Diep Ly, Bastian Fischer, Anika Kleine, Cornelius Knabbe, Isabel Faust-Hinse

**Affiliations:** Institut für Laboratoriums-und Transfusionsmedizin, Herz-und Diabeteszentrum NRW, Universitätsklinik der Ruhr-Universität Bochum, Georgstraße 11, 32545 Bad Oeynhausen, Germany

**Keywords:** Xylosyltransferase, acute senescence, cellular senescence, wound healing, fibrosis, myofibroblast, extracellular matrix

## Abstract

The human xylosyltransferase isoform XT-I catalyzes the initial step in proteoglycan biosynthesis and represents a biomarker of myofibroblast differentiation. Furthermore, XT-I overexpression is associated with fibrosis, whereby a fibrotic process initially develops from a dysregulated wound healing. In a physiologically wound healing process, extracellular matrix-producing myofibroblasts enter acute senescence to protect against fibrosis. The aim of this study was to determine the role of XT-I in acute senescent proto-myofibroblasts. Normal human dermal fibroblasts were seeded in a low cell density to promote myofibroblast differentiation and treated with H_2_O_2_ to induce acute senescence. Initiation of the acute senescence program in human proto-myofibroblasts resulted in a suppression of *XYLT* mRNA expression compared to the control, whereby the isoform *XYLT1* was more affected than *XYLT2*. Moreover, the XT-I protein expression and enzyme activity were also reduced in H_2_O_2_-treated cells compared to the control. The examination of extracellular matrix remodeling revealed reduced expression of collagen I, fibronectin and decorin. In summary, acute senescent proto-myofibroblasts formed an anti-fibrotic phenotype, and suppression of XT-I during the induction process of acute senescence significantly contributed to subsequent ECM remodeling. XT-I therefore plays an important role in the switch between physiological and pathological wound healing.

## 1. Introduction

Cellular senescence is an irreversible form of cell cycle arrest, preventing damaged cells from undergoing proliferation and precluding potential neoplastic transformation. The reasons for cellular damage can be very different and include DNA damage, telomere dysfunction, activation of certain oncogenes, oxidative stress, or chromatin disruption [1,2]. Moreover, there are two signaling pathways that individually or simultaneously trigger the cell cycle arrest. After an acute DNA damage response, the p53-dependent induction of p21^CIP/WAF^ follows, but if the cells are exposed to permanent damage, this could lead to a p38 mitogen-activated protein kinase mediated mitochondrial dysfunction and reactive oxygen species release that activates p16^Ink4a^. Induction of both signaling pathways subsequently leads to activation of the tumor suppressor retinoblastoma protein, causing the cells to initiate permanent cell cycle arrest [3,4]. Senescent cells are no longer able to proliferate, but are still metabolically active and express a senescence-associated secretory phenotype (SASP) [1,5]. The SASP can be very heterogeneous and includes different pro-inflammatory cytokines, chemokines and growth factors, as well as extracellular matrix (ECM)-degrading matrix metalloproteinases (MMPs) [5]. However, the most widely used senescence biomarker is the activity of the senescence associated β-galactosidase (SA β-gal), defined as lysosomal β-gal [6,7]. Under physiological conditions, cellular senescence is not only an important mechanism of tumor suppression [8], but is also crucial during embryonic development [9,10], wound healing [11] or tissue repair [12]. Cellular senescence can be divided into acute and chronic senescence [3]. Acute senescence represents a mechanism of fibroblasts to protect against fibrotic changes during the physiological wound healing process [11]. By contrast, chronic senescence is associated with different age-related diseases, such as glaucoma, osteoarthritis, arteriosclerosis or cancer [13]. In relation to the development and manifestation of fibrotic diseases, cellular senescence is controversial. Induction of cellular senescence in lung fibroblasts mediates fibrotic lung disease in mice [14], whereas senescent-activated stellate cells express an anti-fibrotic phenotype and restrict liver fibrosis in mice [12]. Fibrotic disorders are the result of chronic inflammatory reactions, defined by an excessive deposition of ECM components in different tissues and organs, which often leads to organ malfunction. Fibrotic disorders encompass systemic diseases such as systemic sclerosis, and organ-specific disorders, such as liver, cardiac or pulmonary fibrosis [15,16]. One key step in the pathogenesis of fibrosis is the differentiation of resident fibroblasts to excessive ECM-producing myofibroblasts. The fibroblast to myofibroblast transition can be subdivided into two stages. Fibroblasts first become activated to a proto-myofibroblast phenotype, followed by a second stage completing the cell phenotype transition [17,18]. An important mediator of this differentiation step is, inter alia, transforming growth factor β1 (TGF-β1) [19]. Moreover, an increased proteoglycan (PG) expression is associated with most fibrotic diseases [20,21,22]. The PGs are essential components of the ECM and consist of a core protein and one or more covalently attached glycosaminoglycan (GAG) chains. They have various functions via these covalently bound GAG chains; for example, regulation of the ECM structure, cell adhesion and signaling. Moreover, PGs control the gradients and availability of growth factors, chemokines, cytokines and morphogenes during development and tissue repair [23,24,25]. GAG biosynthesis is initiated by the two tissue-specific expressed xylosyltransferase isoforms XT-I and XT-II, which are encoded by the genes *XYLT1* and *XYLT2*, respectively [26]. These transmembrane type II proteins catalyze the initial step of the PG biosynthesis by transferring xylose from UDP-xylose on specific serine residues of the PG core protein [27,28]. Although the xylosylation of the PG core proteins takes place in the Golgi apparatus [28], most of the XT activity can be determined in the extracellular space [29]. There is no UDP-xylose as a donor substrate in the extracellular space, therefore, the active secretion mechanism could be part of the XT activity regulation, whereby the enzyme is shed from the membrane of the Golgi apparatus by an unknown mechanism that involves a cysteine protease [30,31]. Moreover, the XT-I can be used as a biomarker to identify fibrotic processes in systemic sclerosis [29] or during fibrotic scarring as a biomarker for the myofibroblast differentiation [32]. TGF-β1 [32,33,34], activin A [35], interleukine 1β (IL-1β) [34,36] and miRNAs [37,38] are known mediators of the XT-I regulation.

Although the regulation of XT-I in profibrotic processes is mainly understood, little is known about the XT-I regulation during the physiological wound healing process, especially concerning myofibroblasts entering the acute senescence program. That XT may play a crucial role in cellular senescence was shown by the results of our study in which human embryonal kidney (HEK293) cells, already *XYLT1*-deficient, exhibited reduced proliferation capacity after *XYLT2* mRNA knockdown [39]. Based on this, we detected increased SA β-gal activity in normal human dermal fibroblasts (NHDF) that showed permanently reduced *XYLT1* mRNA expression and XT-I activity using the CRISPR/Cas9 system [40]. To investigate XT-I regulation in acute senescent proto-myofibroblasts during the physiological wound healing process, a cell culture model was established based on the protocols of Masur et al. [41] and Faust et al. [32], in which NHDF were seeded at a low cell density (50 cells/mm^2^) to promote the differentiation into proto-myofibroblasts. Subsequently, acute senescence associated with the physiological wound healing process was induced by treatment with H_2_O_2_, a widely used reagent to induce cellular senescence in different cell types [42,43].

## 2. Materials and Methods

### 2.1. Cell Culture and Hydrogen Peroxide Treatment

NHDF from a 57- and a 55-year-old man, as well as a 50-year-old woman, were purchased from Coriell (Camden, NY, USA). Cells were cultivated as a monolayer culture on hard plastic cell culture dishes with complete medium consisting of Dulbecco’s modified Eagle´s medium supplemented with 10% (*v*/*v*) fetal calf serum (Thermo Fisher Scientific, Waltham, MA, USA), 4 mM L-glutamine and 1% Penicillin-Streptomycin-Amphotericin B solution (100×; PAN Biotech, Aidenbach, Germany) under standardized conditions (37 °C, 5% CO_2_). Medium changes were performed twice a week and fibroblasts were subcultured with 0.05% trypsin (PAN Biotech, Aidenbach, Germany) in Dulbecco’s phosphate buffered saline (PBS; Thermo Fisher Scientific, Waltham, MA, USA) after reaching 90% confluence.

Cells were subcultured in an expansion ratio of 1:3 on 100 × 20 mm plastic cell culture dishes and subsequently seeded at passage 7 in a cell density of 50 cells/mm^2^. After 24 h, cells were incubated for 1 h with complete medium supplemented with 300 µM H_2_O_2_ (Carl Roth, Karlsruhe, Germany) or an appropriate volume of H_2_O (control). Thereafter, cells were washed with PBS and further cultured in complete medium for an experiment-dependent time. For investigations on enzyme activity or protein level, cells were reseeded at a cell density of 50 cells/mm^2^ and cultured for another 18 or 72 h in complete medium.

### 2.2. Trypan Blue Dye Exclusion Assay

The effect of H_2_O_2_ on cell viability was investigated using the trypan blue dye exclusion assay [44]. Cells were cultivated on plastic 12-well cell culture plates. A period of 72 h after the H_2_O_2_ treatment, human proto-myofibroblasts were reseeded and cultured for another 72 h (72 + 72 h). Cells were detached and trypan blue solution (0.4%) was added to the cell suspension at a ratio of 1:1 and the automatically cell-imaging system JuLi Br (NanoEntrek, Seoul, Republic of Korea) was used to calculate the proportion of viable cells. Cell viability was detected for six technical replicates of one biological replicate per experiment (n = 3). Cell viability was calculated as follows:

(1) $$\mathrm{cell}\;\mathrm{viability}\;\left[\%\right]=\frac{\mathrm{total}\;\mathrm{number}\;\mathrm{of}\;\mathrm{viable}\;\mathrm{cells}\;\mathrm{per}\;\mathrm{mL}\;\mathrm{of}\;\mathrm{cell}\;\mathrm{suspension}\;}{\mathrm{total}\;\mathrm{number}\;\mathrm{of}\;\mathrm{cells}\;\mathrm{per}\;\mathrm{mL}\;\mathrm{of}\;\mathrm{cell}\;\mathrm{suspension}}\times 100$$

### 2.3. Bicinchoninic Acid Assay

A bicinchoninic acid assay was used to determine the protein concentration of cell lysates for western blot analysis, the normalization of the XT-I enzyme activity and the SA β-gal enzyme activity. The assay was carried out according to Smith et al. [45] and performed as previously described [46].

### 2.4. Quantitative Senescence-Associated β-Gal Activity Assay

According to the protocol of Gary and Kindell, the β-gal enzyme activity was quantified by measuring the hydrolysis of 4-methylumbelliferyl β-D-galactopyranoside to the spectrophotometrically detectable product 7-hydroxy-4-methylcoumarin [47]. For this experiment, NHDF were seeded on plastic 6-well cell culture plates. 72 h after H_2_O_2_ treatment, human proto-myofibroblasts were lysed or subcultured for another 72 h. The cell layer was lysed with 150 µL lysis buffer (0.2 M sodium phosphate, 0.1 M citric acid, 5 mM CHAPS, 0.5 mM benzamidine, 0.25 mM PMSF, pH 6.0). Cell lysates were stored at −80 °C for at least 24 h. Then, the samples were centrifuged for 5 min at 12,000× *g* and 100 µL of the clarified supernatant was mixed with 100 µL reaction buffer (0.2 M sodium phosphate, 0.1 M citric acid, 300 mM NaCl, 10 mM β-mercaptoethanol, 4 mM MgCl_2_, 1.7 mM 4-methylumbelliferyl-β-D-galactopyranoside, pH 6.0) and incubated for 1 h at 37 °C. The reaction was stopped by adding 600 µL of a 400 mM sodium carbonate solution. A volume of 150 µL reaction mixture was pipetted on a black 96-well plate and three technical replicates were measured for each biological replicate using a Tecan Reader Infinite 200 PRO from Tecan Trading AG (Maennedorf, CHE; excitation: 360 nm, emission: 465 nm, integration: 40 µs). The fluorescence intensity measured corresponded to the β-gal enzyme activity and was normalized to the total protein concentration of the samples determined by the bicinchonic acid assay. The β-gal enzyme activity was indicated relative to the respective donor control.

### 2.5. Quantitative Reverse Transcription Polymerase Chain Reaction (qRT-PCR)

For this experiment, NHDF were seeded on 100 × 20 mm plastic cell culture dishes. Cells were lysed with 254 µL RA1 lysis buffer (Qiagen, Hilden, DE), including 4 µL β- mercaptoethanol, 6 or 72 h after H_2_O_2_ treatment. The RNA extraction, cDNA synthesis and the mRNA expression analysis with the LightCycler 480 Instrument II system (Roche, Basel, CHE) were performed as previously described [35]. These experiments were carried out with three biological and three technical replicates for each donor-derived primary cell culture. The primer sequences, annealing temperatures and primer efficiencies utilized are listed in Table 1. Data were analyzed using the ΔΔCT method considering the qPCR efficiency of the gene of interest and the internal control gene glyceralaldehyd-3-phosphate-dehydrogenase (*GAPDH*), whereupon the values were normalized. The representation of the mRNA expression levels were shown relative to the mean values of the mRNA expressions of the respective donor controls.

### 2.6. XT-I Selective Enzyme Activity Assay by UPLC/ESI-MS/MS

A period of 72 h after H_2_O_2_ treatment, human proto-myofibroblasts were reseeded on 100 × 20 mm plastic cell culture dishes and cultured for another 18 h with complete medium to avoid a normalization error due to the different proliferation capacity of the control and H_2_O_2_-treated cells. Cell culture supernatants were collected to determine the extracellular XT-I enzyme activity, and cell lysates were generated with 250 µL Nonidet P-40 lysis buffer (50 mM TRIS, 150 mM NaCl, 1% NP-40, pH 7.8) per sample for the analysis of intracellular XT-I activity. The samples were centrifuged for 10 min at 10,000× *g* and 4 °C. After centrifugation, the XT-I protein is in the supernatant and the XT-I activity was determined by a XT-I selective ultra-performance liquid chromatography/electrospray ionization tandem mass spectrometric (UPLC/ESI-MS/MS) assay, which was previously described [48]. Samples were diluted 1:5 with UHPLC water, and three technical replicates per biological replicate were analyzed and normalized to the total protein concentration by bicinchoninic acid assay. The quantification of XT-I activity was determined by using specific standards, which contained different concentrations of the xylosylated peptide, and the respective peak areas correlated with the XT-I activity. The XT-I enzyme activities of the samples were calculated relative to the respective donor control.

### 2.7. Immunoblotting

NHDF were seeded on 100 × 20 mm plastic cell culture dishes. 72 h after H_2_O_2_ treatment and based on the respective results of mRNA expression analysis, acute senescent proto-myofibroblasts were either lysed (detection of XT-I) or reseeded and cultured for an additional 72 h (detection of collagen I, fibronectin and decorin). Cell lysis was performed for 10 min on ice with 300 µL RIPA buffer (Thermo Fisher Scientific, Waltham, MA, USA) including 1× protease inhibitor cocktail (100×; Sigma, St. Louis, MO, USA). Extracts were centrifuged for 10 min at 10,000× *g* at 4 °C. The total protein concentrations of the lysates were determined using the bicinchoninic acid assay. All materials for immunoblotting, excluding antibodies, were from Thermo Fisher Scientific (Waltham, MA, USA). An amount of 5 µg of total protein was separated under reducing conditions on 3–8% tris-acetate (analysis of collagen I and fibronectin) or on 8–16% tris-glycine mini protein gels (analysis of XT-I and decorin). Following the electrophoresis, proteins were transferred to a polyvinylidene difluoride membrane and incubated for 1 h in 5% bovine serum albumin (BSA) in TBS-T (150 mM NaCl, 50 mM Tris pH 7.6 and 0.1% Tween-20). Then, membranes were incubated with the following primary antibodies overnight at 4 °C: anti-human collagen I antibody (1:10,000; cat. no. ab138492), anti-human fibronectin antibody (1:5000; cat. no. ab2413), anti-human decorin antibody (1:1000; cat. no. ab181456), anti-human GAPDH antibody (1:1000; cat. no. ab8245; Abcam, Cambridge, UK) and anti-human XYLT1 antibody (1:500; cat. no. PA5-67627; Thermo Fisher Scientific, Waltham, MA, USA). After washing with TBS-T, membranes were probed for 1 h with diluted horseradish peroxidase conjugated secondary antibodies goat anti-rabbit IgG H&L HRP or goat anti-mouse IgG H&L HRP (1:5000; cat. no. ab97051 and ab6789, respectively; Abcam, Cambridge, UK). The chemiluminescence reagent Super Signal West Pico PLUS was used to detect immunolabeling with the gel documentation system Fusion SL (PeqLab, Erlangen, DE). Image J was used for the quantification of the band intensities. Protein expressions were normalized to GAPDH and presented relative to the respective donor control. Complete blot images can be found in the Supplementary Material (Figures S4–S7).

### 2.8. Immunofluorescence

NHDF were seeded on 100 × 20 mm plastic cell culture dishes. A period of 72 h after H_2_O_2_ treatment, acute senescent proto-myofibroblasts were reseeded on glass cover slips in a 12-well cell culture plate, coated with 5 µg/cm^2^ collagen type I from rat tail (ibidi, Gräfelfing, DE) and cultured for an additional 72 h. Cells were fixed for 30 min with a 4% phosphate buffered formaldehyde solution (Carl Roth, Karlsruhe, DE) and permeabilized for 20 min with 0.1% Triton X-100 in PBS. Then, cells were incubated for 1 h with a blocking solution consisting of 10% goat serum, 5% BSA, 0.3 M glycine and PBS- T (0.1% Tween-20 in PBS). The primary antibodies against human p16^INK4a^ and p21^CIP/WAF^ were diluted in 1% BSA in PBS-T and probed overnight at 4 °C (1:200; cat. no. ab108349 and ab109520, respectively; Abcam, Cambridge, UK). The secondary Alexa Fluor 555-conjugated goat anti-rabbit IgG antibody was diluted in 1% BSA in TBS-T and the samples were incubated for 1 h in the dark (1:400; cat. no. ab150078; Abcam, Cambridge, UK). DAPI (1:200 in PBS; Abcam, Cambridge, UK) was used for the cell nuclei staining. Glass cover slips were mounted with ProLong Diamond Antifade Mountant (Thermo Fisher Scientific, Waltham, MA, USA) and visualized with an immunofluorescence microscope (Nikon, Tokyo, JPN). Protein expression was quantified using ImageJ by determining the corrected total cell fluorescence (CTCF). Six cells were analyzed for each biological replicate and the results were shown relative to the respective donor control (relative CTCF).

(2) CTCF=Integrated density−(area of selected cell × mean fluorescence of background readings) 

### 2.9. Statistical Analysis

GraphPad Prism 9 (GraphPad Software, La Jolla, CA, USA) was used for the statistical analysis and to create the graphics of this study. The statistical method used for the comparisons between experimental conditions was the nonparametric two-tailed Mann–Whitney U test. Data are presented as mean values ± standard error of the mean (SEM), and *p* ≤ 0.05 was considered statistically significant. *p* values are shown as asterisks and horizontal lines connect the bars being compared.

## 3. Results

### 3.1. H_2_O_2_-Treatment of Human Proto-Myofibroblasts Induces Acute Senescence

In order to study the role of XT-I in acute senescence during the physiological wound healing process, we established a protocol for senescence induction by H_2_O_2_ treatment, a widely used method to generate stress-induced premature senescence in different cell types [41,43]. Hydrogen peroxide belongs to the reactive oxygen species and can lead to the induction of cellular senescence during a physiological cutaneous wound healing process [11]. Furthermore, cultivating NHDF in a cell density of 50 cells/mm^2^ promotes myofibroblast differentiation [41]. The combination of culturing NHDF in a low cell density on a hard tissue supplement and the H_2_O_2_ treatment should mimic the microenvironment of a physiological wound healing process in which human myofibroblasts enter acute senescence or apoptosis to protect against fibrosis. We used a quantitative SA β-gal enzyme activity assay to detect senescence induction in human proto-myofibroblasts after H_2_O_2_ treatment [47]. We evaluated the effect of different H_2_O_2_ concentrations between 100 and 500 µM and different incubation times to determine the lowest H_2_O_2_ concentration and cultivation time to induce acute senescence in human proto-myofibroblasts. Based on this preliminary work, an H_2_O_2_ concentration of 300 µM and an incubation time of 1 h were used for the subsequent experiments in this study.

**Figure 1:**
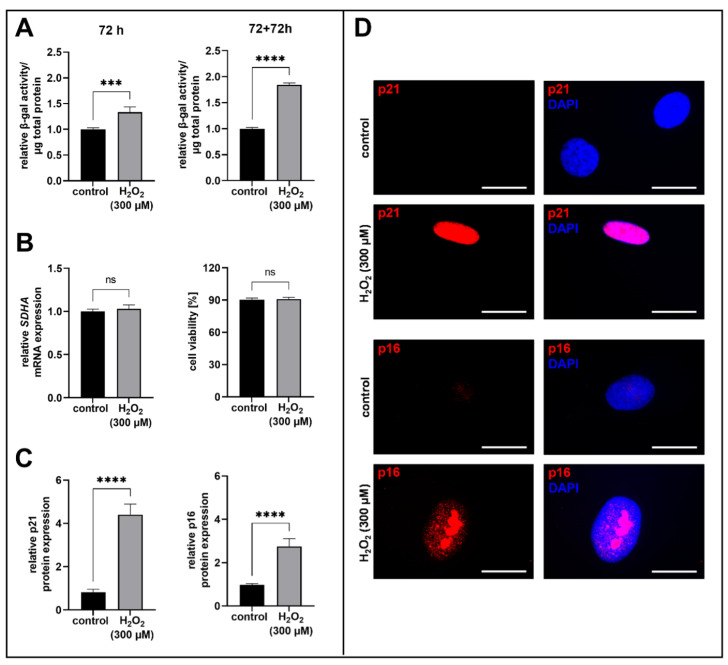
Quantification of H_2_O_2_-induced acute senescence in human proto-myofibroblasts. After a cultivation time of 24 h, cells were treated for 1 h with 300 µM H_2_O_2_ or H_2_O (control). (**A**) Acute senescence of human proto-myofibroblasts was detected by the determination of SA β-galactosidase activity after 72 h or after reseeding and an additional cultivation time of 72 h (72 + 72 h). The spectrophotometrically measurement of the fluorescence intensity of 4-methylumbelliferyl-β-D-galactopyranoside was detected by using the Tecan Reader Infinite 200 PRO (excitation: 360 nm, emission: 465 nm, integration: 40 µs). The values shown are means ± SEM for three biological and three technical replicates per experiment (n = 3) and were calculated relative to the respective control. (**B**) The cell viability was investigated by measuring the succinate dehydrogenase complex flavoprotein subunit A (*SDHA*) mRNA expression level at 6 h by quantitative real-time PCR using the glyceralaldehyd-3-phosphatedehydrogenase (*GAPDH*) mRNA expression level for expression normalization (left panel) and by trypan blue dye exclusion assay after 72 + 72 h (right panel). Viable cells were counted by using the automatic cell imaging system JuLi Br. The values shown are means ± SEM for three or six technical replicates of three or one biological replicate per experiment (n = 3) and were calculated relative to the respective control. (**C**) Quantification of the p21 and p16 protein expression by immunofluorescence analysis. The corrected total cell fluorescence was determined by using ImageJ. The values are means ± SEM for three biological replicates per experiment (n = 3) and were calculated relative to the respective control. (**D**) Representative images of p21 and p16 immunofluorescence staining (red). The cell nuclei were counterstained with DAPI (blue). Scale bars are 20 µM. Mann-Whitney U test: not significant (ns), *p* < 0.001 (***), *p* < 0.0001 (****).

The initiation of acute senescence in human proto-myofibroblasts was verified at the protein level after 72 h and after an additional cultivation time of 72 h (72 + 72 h time point). These two harvest time points after H_2_O_2_ treatment were chosen so that further analyses of the transcriptome and proteome of the human myofibroblasts would reflect the permanent expression changes due to the induction of acute senescence. Moreover, the start of the acute senescence induction process was identified by the measurement of p21 and p16 mRNA expression 6 h after H_2_O_2_ treatment. After 72 h and 72 + 72 h, the relative SA β-gal activity significantly increased in the H_2_O_2_-treated human proto-myofibroblasts compared to the control (1.3-fold and 1.8-fold, *p* < 0.001 and *p* < 0.0001, respectively; Figure 1A). The cell viability was determined at the 6 h time point by measuring the mRNA expression of the succinate dehydrogenase complex flavoprotein sub-unit A (Figure 1B, left panel) and at the 72 + 72 h time point by using a trypan blue dye exclusion assay (Figure 1B, right panel). The acute senescence induction of human proto-myofibroblast by H_2_O_2_ treatment does not affect the cell viability at both time points. In addition to the SA β-gal activity, the mRNA and protein expressions of p21 and p16 were determined as additional senescence biomarkers by quantitative real-time PCR and immunofluorescence staining. Compared with the control, *p21* and *p16* mRNA expressions were significantly increased in the H_2_O_2_-treated human myofibroblasts after 6 h (1.9-fold and 1.7-fold, *p* < 0.0001 and *p* < 0.05, respectively; Supplementary Material Figure S1). Regarding the relative protein expression of p21, a significant 4.2-fold increase was demonstrated after a cultivation time of 72 + 72 h for the H_2_O_2_-treated human proto-myofibroblasts in comparison to the control (*p* < 0.0001; Figure 1C). The p16 protein expression also significantly increased 3.2-fold in the H_2_O_2_-treated cells in comparison to the control (*p* < 0.0001; Figure 1C). Fluorescence images of p21 and p16 from one donor-derived primary cell culture are representatively shown in Figure 1D and overview images at a lower magnification are shown in the Supplementary Material (Figures S2 and S3).

The H_2_O_2_ treatment resulted in a successful induction of acute senescence in human proto-myofibroblasts, although cell viability was not affected.

### 3.2. Suppression of XT Expression in Acute Senescent Proto-Myofibroblasts

Cellular senescence is an essential mechanism for maintaining tissue homeostasis. It could be demonstrated in previous studies that the transition of myofibroblasts into a senescence status is a preprogrammed component of the wound healing mechanism to self-limit the transition from a physiological to a pathophysiological wound healing process; for example, tissue fibrosis [11,49]. The regulation of XT-I has so far only been investigated in relation to diseases characterized by an altered PG metabolism [31]; therefore, we wanted to examine the role of XT-I during the transition of proto-myofibroblasts into an acute senescence status. After acute senescence had been successfully induced in human proto-myofibroblasts, we analyzed the effect of acute senescence on *XYLT* mRNA expression 72 h after H_2_O_2_ treatment. In addition, the *XYLT* mRNA expression was measured 6 h after H_2_O_2_ treatment, because the *XYLT1* is an early response gene [35].

**Figure 2:**
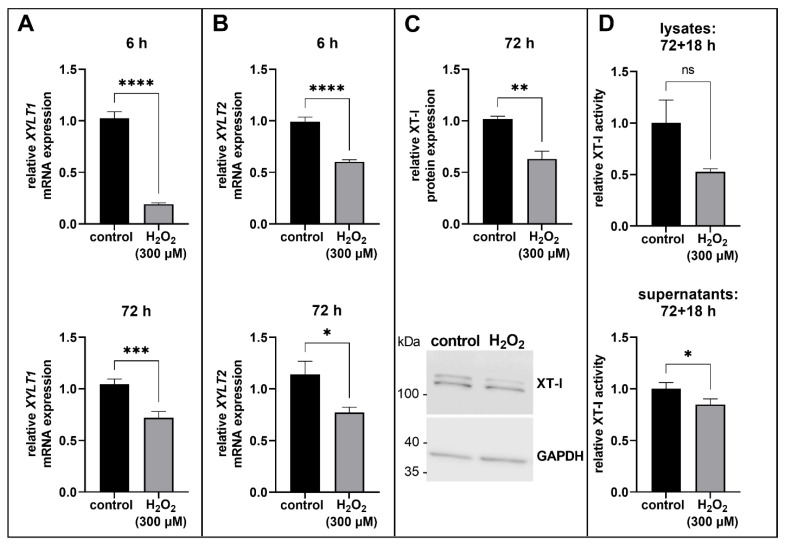
Investigation of the *XYLT* mRNA and XT-I protein expression level in acute senescent proto-myofibroblasts. After 24 h of cultivation, cells were treated for 1 h with 300 µM H_2_O_2_ or H_2_O (control). *XYLT*1 (**A**) and XYLT2 (**B**) mRNA expression levels were analyzed after 6 or 72 h of cultivation by quantitative real-time PCR and were normalized on *GAPDH* mRNA expression level. The XT-I protein expressions in cell lysates were detected via immunoblotting 72 h after H_2_O_2_ treatment and were normalized on GAPDH protein expression. Quantification was determined using ImageJ (**C**). The intra- and extracellular XT-I enzyme activities were determined after 72 + 18 h by a XT-I-selective mass spectrometric assay (UPLC/ESI-MS/MS assay) and were normalized on total protein concentrations (**D**). The values shown are means ± SEM for three biological and three (**A**,**B**,**D**) or one (**C**) technical replicate per experiment (n = 3) and calculated relative to the respective control. Mann-Whitney U test: not significant (ns), *p* < 0.05 (*), *p* < 0.01 (**), *p* < 0.001 (***), *p* < 0.0001 (****).

We found that *XYLT1* and *XYLT2* gene expression levels significantly decreased after an incubation time of 6 h (0.2 and 0.6-fold, respectively, both *p* < 0.0001; Figure 2A,B) and 72 h (0.7 and 0.6-fold, *p* < 0.001 and *p* < 0.05, respectively; Figure 2A,B). Based on the early response of *XYLT1* to the H_2_O_2_ treatment, as well as the fact that only XT-I is related to fibrotic diseases and is used as a myofibroblast marker, the further experiments on protein level were performed 72 h after H_2_O_2_ treatment and only with XT-I. The determination of the XT-I protein expression was carried out by immunoblotting. We determined a significantly decreased XT-I protein expression in the H_2_O_2_-treated cells in comparison to the control (0.6-fold, *p* < 0.01; Figure 2C). A representative complete original blot image for the quantification of the XT-I protein expression is shown in the Supplementary Material (Figure S4). Moreover, the enzyme activity of XT-I was measured by a XT-I selective mass spectrometric assay. Then, 72 h after H_2_O_2_-treatment, cells were incubated for a further 18 h to avoid a normalization error due to the different proliferation capacity of the control and H_2_O_2_-treated cells. Thereby a cultivation time of 18 h is enough to detect extracellular XT-I activity, too. The evaluation of the XT-I selective mass spectrometric assay confirmed the results of the mRNA and protein expression analysis of XT-I. The intracellular XT-I activity was not significantly decreased (0.5-fold, *p* = 0.1135; Figure 2D) and the extracellular XT-I activity was significantly reduced in the H_2_O_2_-treated proto-myofibroblasts (0.9-fold, *p* < 0.05; Figure 2D).

We could demonstrate, through our experiments, that the *XYLT* mRNA expression was suppressed in acute senescent human proto-myofibroblasts, whereby *XYLT1* was more affected than *XYLT2*. Moreover, the XT-I protein expression and enzyme activity were also decreased in comparison to the control.

### 3.3. ECM Remodeling in Acute Senescent Proto-Myofibroblasts

Proto-myofibroblasts excessively produce collagen I (*COL1A1*) and fibronectin (*FN*) during wound healing or fibrotic processes; therefore, we analyzed the expression of these two ECM components at gene and protein levels. In addition, we analyzed decorin (*DCN*), an end product of the PG biosynthesis, to validate the results of the XT-I protein expression and enzyme activity analysis.

**Figure 3:**
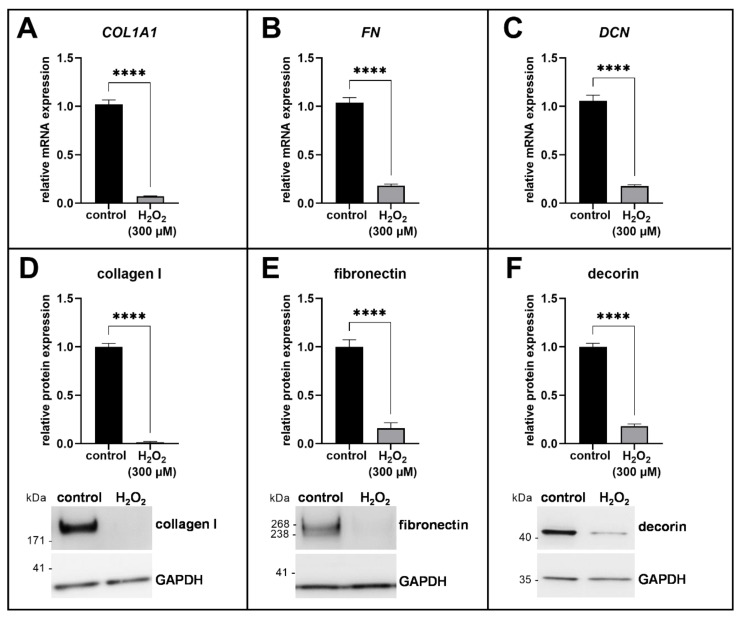
Analysis of ECM remodeling in acute senescent proto-myofibroblasts. After 24 h of cultivation, cells were treated for 1 h with 300 µM H_2_O_2_ or H_2_O (control). Relative *COL1A1* (**A**), *FN* (**B**) and *DCN* (**C**) mRNA expression levels were analyzed after 72 h of cultivation by quantitative real-time PCR and were normalized on *GAPDH* mRNA expression level. The protein expression levels of collagen I (**D**), fibronectin (**E**) and decorin (**F**) were detected after a cultivation time of 72 + 72 h via immunoblotting and normalized on GAPDH protein expression. Quantification was determined by using ImageJ. Values shown are means ± SEM for three biological and three (**A**–**C**) or one (**D**–**F**) technical replicate per experiment (n = 3) and calculated relative to the respective control. Mann-Whitney U test: *p* < 0.0001 (****).

We evaluated the ECM expression profile of H_2_O_2_-treated human proto-myofibroblasts on the mRNA expression level by quantitative real-time PCR. After 72 h of cultivation, we showed a significant decrease in the mRNA expression of *COL1A1*, *FN* and *DCN* in the H_2_O_2_-treated human myofibroblasts compared to the control (0.1-fold for each of the genes, *p* < 0.0001; Figure 3A–C). In accordance with the mRNA expression data of the ECM components, we determined the protein expression 72 h after reseeding by immunoblotting (Figure 3D–F). The protein expression of collagen I was almost undetectable (0.01-fold, *p* < 0.0001; Figure 3D) and the expression of fibronectin and decorin was also significantly decreased in the H_2_O_2_-treated cells compared to the control (0.2-fold for both, *p* < 0.0001; Figure 3E,F). The representative complete original blot images for the quantification of the protein expressions are shown in the Supplementary Material (Figures S5–S7).

The results of the ECM expression analysis were in accordance with the data of XT-I protein expression and enzyme activity. The induction of acute senescence in human proto-myofibroblasts resulted in the formation of an anti-fibrotic phenotype characterized by a strongly down-regulated ECM synthesis [16,29].

### 3.4. The SASP of Acute Senescent Proto-Myofibroblasts

Specific members of the SASP were selected and analyzed at the gene expression level to obtain clues to the underlying signaling pathways that might lead to XT-I regulation in acute senescent proto-myofibroblasts. Regarding XT-I regulation in acute senescent proto-myofibroblasts, TGF-β1 (*TGFB1*) and IL-1β (*IL1B*) were selected because both molecules have already been shown to have a regulatory effect on XT-I, although this is related to fibrotic processes. In addition, interleukin-8 (*CXCL8*) was selected as another classic representative of the SASP. The gene and protein expression of collagen I was significantly reduced in acute senescent proto-myofibroblasts; therefore, fibroblast collagenase MMP1 was analyzed on mRNA expression level to further investigate the underlying regulatory mechanism.

**Figure 4:**
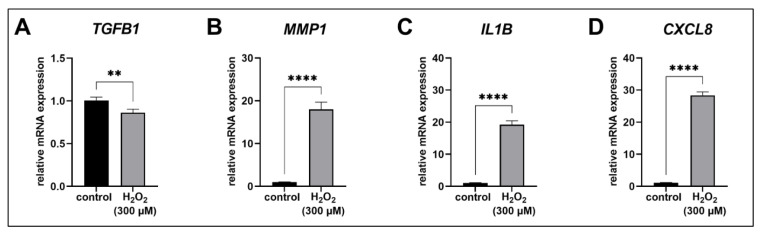
Expression analysis of the SASP in acute senescent proto-myofibroblasts. After 24 h of cultivation, cells were treated for 1 h with 300 µM H_2_O_2_ or H_2_O (control). Relative *TGFB1* (**A**), *MMP1* (**B**), *IL1B* (**C**) and *CXCL8* (**D**) mRNA expression levels were determined after 72 h of cultivation by quantitative real-time PCR and were normalized on *GAPDH* mRNA expression level. Values shown are means ± SEM for three biological and three technical replicates per experiment and were calculated relative to the respective control. Mann-Whitney U test: *p* < 0.01 (**) and *p* < 0.0001 (****).

The mRNA-expression of *TGFB1* was only slightly affected 72 h after H_2_O_2_ treatment, which was detected by a significant decrease in the H_2_O_2_-treated human proto-myofibroblasts in comparison to the control (0.9-fold, *p* < 0.01; Figure 4A). By contrast, the H_2_O_2_ treatment of the cells led to a significant increase in *MMP1*, *IL1B* and *CXCL8* mRNA expression compared to the control (18.0, 18.2 and 25.9-fold, respectively, *p* < 0.0001; Figure 4B–D).

The acute senescent proto-myofibroblasts expressed a SASP characterized by increased mRNA expressions of *MMP1*, *IL1B* and *CXCL8*. The *TGFB1* mRNA expression was only marginal altered; therefore, TGF-β1 does not appear to have a regulatory effect on XT-I expression in acute senescent proto-myofibroblasts.

## 4. Discussion

Wound healing is an essential physiological process that restores tissue homeostasis and function after injury. By contrast, as a part of a dysregulated wound healing process, there is an accumulation of persistent myofibroblasts and a permanently increased ECM synthesis in the tissue, which is associated with diseases such as systemic sclerosis or liver fibrosis [50,51]. Moreover, during the wound healing process, the transition of myofibroblasts into a senescent state is described as a self-limiting process to protect against fibrosis [11]. Although there have been many studies investigating the regulatory mechanisms of XT-I in relation to fibrotic diseases, little is known about the role of XT-I during the transition of myofibroblasts into an acute senescence state, such as occurs in a physiological wound healing process. Analysis of XT-I regulation in acute senescent proto-myofibroblasts may provide insights into whether XT-I could function as a novel therapeutic target in dysregulated senescence induction during the wound healing process.

In order to investigate XT-I regulation in acute senescence, NHDF were cultured in a low cell density on a hard tissue supplement with FCS-containing medium to promote myofibroblast differentiation [32,41] and acute senescence was induced by a treatment with 300 µM H_2_O_2_ for 1 h. The senescence induction by H_2_O_2_ was verified by a cell viability assay, because the induction of senescence or apoptosis is dependent on cell type and H_2_O_2_ concentration [42,43,52]. The H_2_O_2_ treatment of proto-myofibroblasts resulted in increased SA β-gal activity, which is considered to be a senescence marker [47], and is associated with increased lysosomal protein concentration, increased β-gal activity and the accumulation of lysosomes [6,53]. Furthermore, increased p21 and p16 protein expression in the H_2_O_2_-treated human proto-myofibroblasts confirmed that the cells were in a permanent cell cycle arrest and, thus, had entered a senescent state. At the 6 h time point, induction of the acute senescence program was identified by increased *p21* and *p16* mRNA expression levels in the H_2_O_2_-treated human proto-myofibroblasts. The H_2_O_2_-induced activation of the p53/p21-dependent pathway to initiate cellular senescence has also been demonstrated in the literature in vascular endothelial cells [54].

In our study, we were able to show, for the first time, a downregulation of the *XYLT* mRNA expression in human acute senescent proto-myofibroblasts, whereby *XYLT1* was more affected than *XYLT2*. Further investigation of XT-I coincided with the results of the mRNA expression analysis and showed reduced XT-I protein expression and enzyme activity in acute senescent proto-myofibroblasts compared to the control. Accordingly, together with the data of the study by Fischer et al., in which *XYLT1*-deficient NHDF showed an increased SA β-gal activity and a decreased expression of ECM components, such as collagen I [40], the XT-I seems to play an important role not only in fibrotic ECM remodelling processes, but also in the late stage of the physiological wound healing process when myofibroblasts enter acute senescence to protect against fibrosis.

In the literature, a reduced mRNA expression in connection with H_2_O_2_ treatment has only been described so far for the isoform XT-II [55]. In addition, the study by Nakayama and colleagues used the keratinocyte cell line HaCaT instead of human dermal fibroblasts and investigated H_2_O_2_ as a potential mediator of the transcriptional regulation of heparan sulfate biosynthesis, rather than as an inducer of cellular senescence [55]. By contrast, suppressed *XYLT1* mRNA expression was detected by Jin et al. 12 h after ultra violet (UV) irradiation of human dermal fibroblasts, examining the glycosylation status of biglycan in UV-damaged skin [56]. However, the treatment of human dermal fibroblasts with UV irradiation appears to induce a different form of cellular senescence compared to a treatment with H_2_O_2_. In the study of Jin et al., human dermal fibroblasts were shown to have only slightly reduced *XYLT1* mRNA expression 24 h after UV irradiation, whereas *XYLT2* mRNA expression was significantly increased three-fold [56].

Although mRNA expressions of both XT isoforms were strongly reduced immediately after H_2_O_2_ treatment, no further analysis at the protein level was performed for the isoform XT-II because only XT-I is a biomarker for myofibroblast differentiation in skin fibrosis [32]. The results of XT-I protein expression and enzyme activity analysis coincided with the *XYLT1* mRNA expression data and showed a suppression of the XT-I. The decreased XT-I activity was predominantly intracellularly detected, as only a small fraction of the total XT amount was secreted at the 18 h time point of cell harvest. The accumulation of XT in the extracellular space over time as a result of a regulatory secretion mechanism has been shown several times [29,35,46]. The determined intracellular XT-I activity reduction had no significance because the donors used in this study had different sensitivities to H_2_O_2_ treatment and so the relative extent of XT-I activity reduction per donor differs from each other. We have considered that fact during the establishment of the experimental design by choosing a H_2_O_2_ concentration and a treatment duration at which increased senescence induction was detectable for all three donors, while no apoptosis was induced. Therefore, depending on the extent to which senescence was induced in the individual donor cultures, a different intensity of XT-I activity regulation was also detected.

The SASP and ECM expression pattern of H_2_O_2_-induced acute senescent proto-myofibroblasts were analyzed to identify possible targets that may be involved in XT-I regulation during acute senescence. The H_2_O_2_ treatment can lead to increased expression of the transcription factors activator protein 1 (AP-1) and nuclear factor kappa B (NF-_Κ_B), inducing the formation of a cell specific SASP [57,58]. It is possible that the regulation of XT-I in acute senescent proto-myofibroblasts occurs through the proinflammatory SASP member IL-1β [5], whose mRNA expression, in addition to that of IL-8 (*CXCL8*), was significantly increased in the H_2_O_2_-treated cells. IL-1β led to the suppression of XT-I in cartilage tissue of late stage osteoarthritis patients [36]. Moreover, IL-1β-mediated suppression of XT-I in human primary chondrocyte cells was shown to be caused by the enhanced binding of repressor-specific protein 3 (Sp3) to the specific protein 1 (Sp1) binding sites of the *XYLT1* promoter region [59].

Furthermore, the *XYLT1* promoter region has binding sites for AP-1 in addition to binding sites for the transcription factors Sp1, Sp3 and Kruppel-like factor 4 (KLF4) [37,59,60], therefore, XT-I regulation through the increased binding of AP-1 to the *XYLT1* promoter region is conceivable. Reduced *XYLT1* and XT-I expression was detected after the treatment of human primary chondrocytes with 29-kDa amino-terminal fibronectin fragments in a study by Hwang and colleagues [61]. The fibronectin fragments were found to suppress the binding of Sp1 to the *XYLT1* promoter region and enhanced the binding of Sp3 and AP-1. Moreover, XT-I suppression was reversed by the inhibition of NF-_Κ_B and the mitogen-activated protein kinase [61]. The H_2_O_2_-treated NHDF in our study exhibited reduced fibronectin protein expression; thus, FN fragments may also have resulted from protease-mediated degradation and led to decreased XT-I expression.

The hypothesis that XT-I regulation in acute senescence might occur via AP-1 can be supported by the increase in *MMP1* mRNA expression and greatly reduced collagen I expression detected in human proto-myofibroblasts treated with H_2_O_2_. In human dermal fibroblasts, a c-Jun/AP-1-mediated ROS-induced increase in expression of the matricellular protein CCN1, which has a negative-regulatory effect on collagen I and also activates *MMP1* mRNA expression, has already been demonstrated [62,63]. The increase of *MMP1* mRNA expression detected in the acute senescent myofibroblasts and the strongly decreased expression of ECM components were also detected in senescent cells primarily derived from human activated hepatic stellate cells [12] and in the granulation tissue of wildtype mice in comparison to *Ccn1^dm^*^/*dm*^ mice [11]. In both studies, the effect of the cellular senescent phenotype on the surrounding tissue was characterized as anti-fibrotic [11,12], whereby a direct comparison to the data of our study is difficult, because both studies used a mice model.

Although decorin has an anti-fibrotic effect through its neutralizing effect on TGF-β1 [64], the decreased decorin expression in the H_2_O_2_-treated human proto-myofibroblasts can be explained by the increase of *MMP1* mRNA expression. The protease MMP-1 is not only responsible for the degradation of collagen I, but also for the degradation of PGs or the release of membrane-bound IL-1β [65]. Accordingly, *MMP1*, as a component of the SASP, might be involved in the increased expression of *IL1B* and the decreased decorin expression in acute senescent human proto-myofibroblasts. A decreased decorin expression after senescence induction can be further demonstrated by the detected suppression of XT-I protein expression and enzyme activity or by a study of Mavrogonatou et al., in which decorin expression was also decreased in human mammary stromal fibroblasts after premature senescence status was induced by ionizing radiation [66].

Furthermore, XT-I suppression could be mediated by increased KLF4 expression. Several studies have already shown that KLF4 can initiate cell cycle arrest in vitro [67,68]. The primary mechanism behind this was elucidated by treating cells with DNA-damaging agents. The KLF4 was shown to trigger transactivation via binding to Sp1-like *cis* elements of the proximal region of the *CDKN1A* promoter, recruiting p53 and further activating the transcription of the p21-coding gene [69,70]. KLF4-mediated induction of permanent cell cycle arrest would be conceivable because this work also involved treatment with a DNA-damaging agent and detected increased p21 protein expression. Regarding the XT-I expression, KLF4 has a suppressive effect that was demonstrated in previous work by the discovery of a novel XT-regulating miR-145/KLF4 pathway [37]. The latter provides a better understanding of the mechanism responsible for fibrotic induction of XT-I in systemic sclerosis patients. In the study by Ly et al., decreased *KLF4* mRNA expression was detected in fibroblasts from systemic sclerosis patients compared with control fibroblasts, along with increased *XYLT1* mRNA expression and increased XT-I activity [37]. The decreased *KLF4* mRNA expression can be explained by a TGF-β1-induced increase of miR-145 expression, whose direct target includes *KLF4* and leads to *KLF4* mRNA degradation [37]. Thus, increased KLF4 expression, as present after treatment with DNA-damaging agents, could explain the detected reduction in *XYLT1* and XT-I expression by KLF4 binding to the corresponding binding sites of the *XYLT1* promoter region and thereby repressing its transcription.

## 5. Conclusions

Our study demonstrated the suppression of XT, particularly XT-I, in the early phase of acute senescent induction in human proto-myofibroblasts, together with reduced ECM synthesis, for the first time. An increased *XYLT1* mRNA expression and XT-I activity are associated with fibrotic processes [32,33,34], and the latter initially arise from dysregulated wound healing [71]; therefore, XT-I appears to have an important function in the switch between physiological and pathological wound healing. Induction of acute senescence resulted in the formation of a SASP characterized by an increased expression of *MMP1*, *IL1B* and *CXCL8*. Thus, the suppression of XT-I in acute senescent proto-myofibroblasts could have been mediated by either IL-1β or the SASP-associated transcription factors AP-1 and KLF4. However, further investigations are needed to identify the underlying signaling pathways of XT-I regulation in acute senescent proto-myofibroblasts. The results of this study also support the notion that XT-I is a potential target for anti-fibrosis therapy. A controlled induction of acute senescence in fibrotic wound healing processes could counteract the manifestation of fibrotic diseases, whereby, not only XT-I, but also ECM synthesis would be suppressed. In order to consider such a therapeutic option, we will perform further experiments, including paracrine effects on surrounding untreated cells.

## Figures and Tables

**Table 1:** Primer pairs used for the qRT-PCR and the respective gene names, annealing temperatures and qPCR efficiencies.

Gene	Primers	T_A_ [°C]	Efficiency
***hCDKN1A***	5′-GCTTCATGCCAGCTACTTCC-3′ 5′-CCCTTCAAAGTGCCATCTGT-3′	66	2.00
***hCDKN2A***	5′-ACCAGAGGCAGTAACCATGC-3′ 5′-AAGTTTCCCGAGGTTTCTCAG-3′	66	2.00
***hCOL1A1***	5′-GATGTGCCACTCTGACT-3′ 5′-GGGTTCTTGCTGATG-3′	63	1.74
***hCXCL8***	5′-GAACTGAGAGTGATTGAGAGTGGA-3′ 5′-CTCTTCAAAAACTTCTCCACAACC-3′	63	1.88
***hDCN***	5′-CCTTCCGCTGTCAATG-3′ 5′-GCAGGTCTAGCAGAGTTG-3′	63	1.76
***hFN***	5′-CCCAGGGAAGATGTAGA-3′ 5′-CTCTTCCCGAACCTTATG-3′	63	2.00
***hGAPDH***	5′-AGGTCGGAGTCAACGGAT-3′ 5′-TCCTGGAAGATGGTGATG-3′	59	1.83
***hIL1B***	5′-ACAGATGAAGTGCTCCTTCCA-3′ 5′-GTCGGAGATTCGTAGCTGGAT-3′	63	1.94
***hMMP1***	5′-AGAAACACAAGAGCAAGATGTG-3′ 5′-TGGCGTGTAATTTTCAATCCTGT-3′	63	1.85
***hSDHA***	5′-AACTCGCTCTTGGACCTG-3′ 5′-GAGTCGCAGTTCCGATGT-3′	66	2.00
***hTGFB1***	5′- GCGATACCTCAGCAACC-3′ 5′- ACGCAGCAGTTCTTCTCC-3′	59	1.95
***hXYLT1***	5′-TGTGACCTTCTCCACAGACG-3′ 5′-CCACGATGTGCTTGTACTGG-3′	63	2.00
***hXYLT2***	5′-ACACAGATGACCCGCTTGTGG-3′ 5′-TTGGTGACCCGCAGGTTGTTG-3′	63	1.95

## Data Availability

Not applicable.

## Supplementary Materials

The following supporting information can be downloaded at: https://www.mdpi.com/article/10.3390/biomedicines11020460/s1, Figure S1: Determination of the *p21* and *p16* mRNA expression in human proto-myofibroblasts 6 h after H_2_O_2_-treatment.; Figure S2: Representative overview images of p21 immunofluorescence staining in acute senescent proto-myofibroblasts.; Figure S3: Representative overview images of p16 immunofluorescence staining in acute senescent proto-myofibroblasts.; Figure S4: Analysis of the XT-I protein expression in acute senescent proto-myofibroblasts.; Figure S5: Analysis of collagen I protein expression in acute senescent proto-myofibroblasts.; Figure S6: Analysis of fibronectin protein expression in acute senescent proto-myofibroblasts.; Figure S7: Analysis of decorin protein expression in acute senescent proto-myofibroblasts.
