# Supplementary material for: Human Xylosyltransferase I—An Important Linker between Acute Senescence and Fibrogenesis
Source: Biomedicines. 2023 Feb 4;11(2):460. doi: 10.3390/biomedicines11020460 (PMC9953725; doi:10.3390/biomedicines11020460)
Supplement: Supplementary file 1 [file biomedicines-11-00460-s001.zip › biomedicines-2161795-supplementary.pdf]

Supplementary Materials

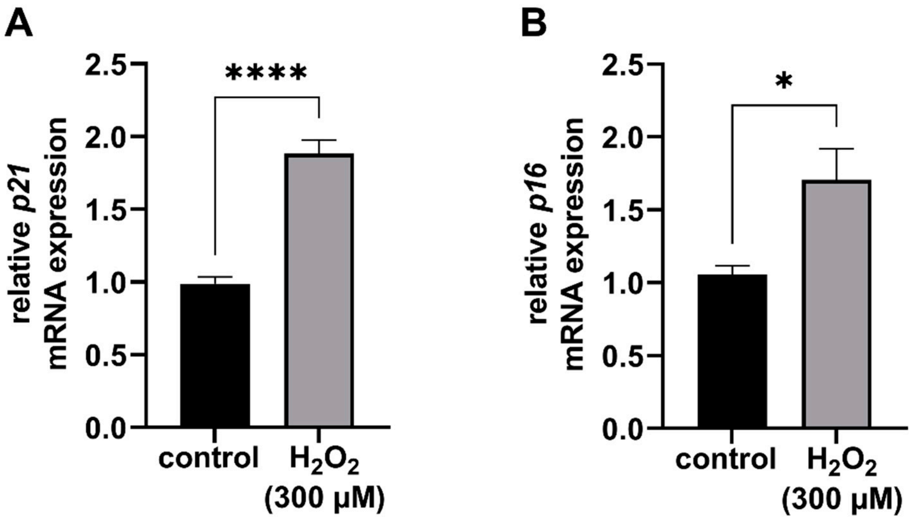

Figure S1. Determination of the *p21* (**A**) and *p16* (**B**) mRNA expression levels in human proto-myofibroblasts 6 h after H_2O_2-treatment. Mann-Whitney U test: $p < 0.05$ (*) and $p < 0.0001$ (****).

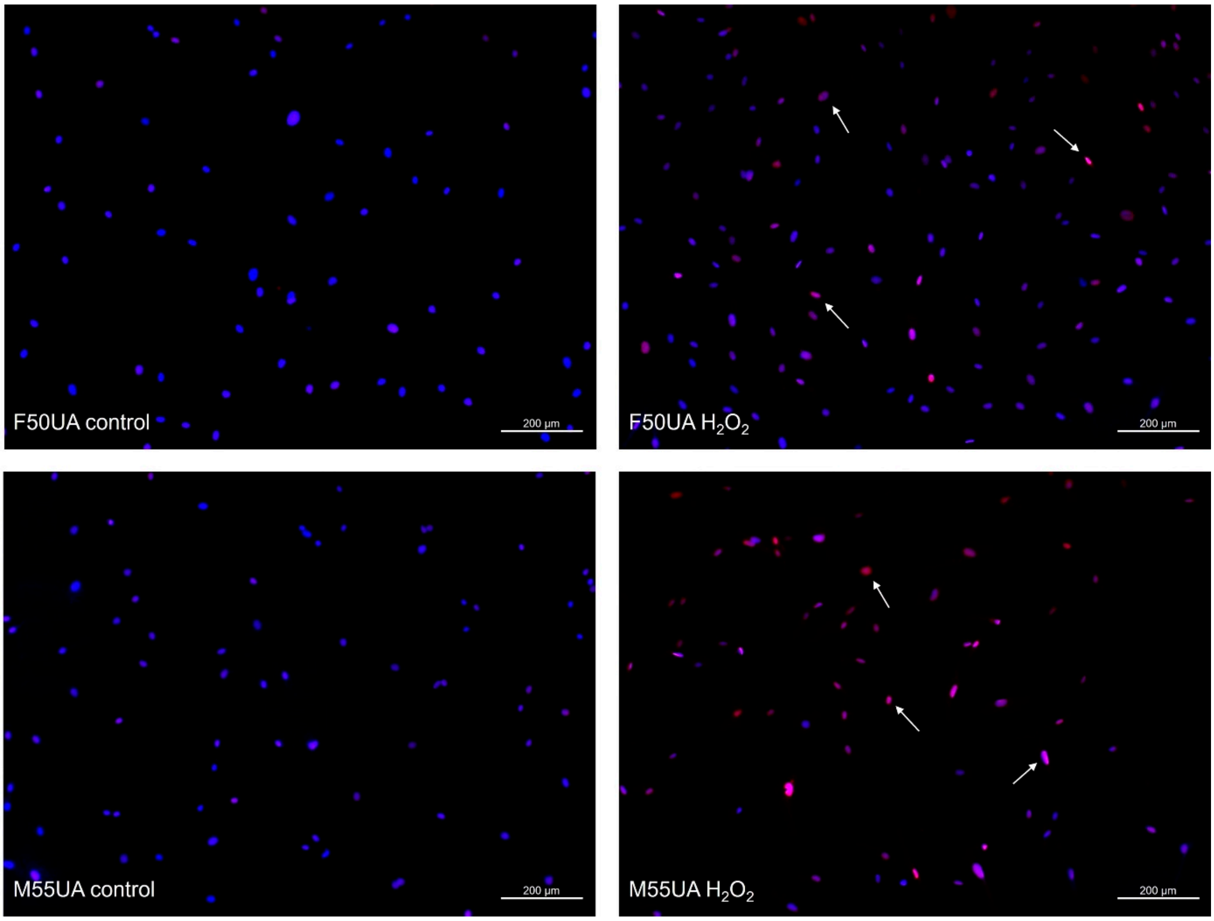

Figure S2. Representative overview images of p21 immunofluorescence staining in acute senescent proto-myofibroblasts. The white arrows in the figure mark exemplary p21 positive cells (red). The cell nuclei were

counterstained with DAPI (blue). Three biological replicates per donor and condition were applied. The images were taken with a 100x magnification (scale bar: 200 µM).

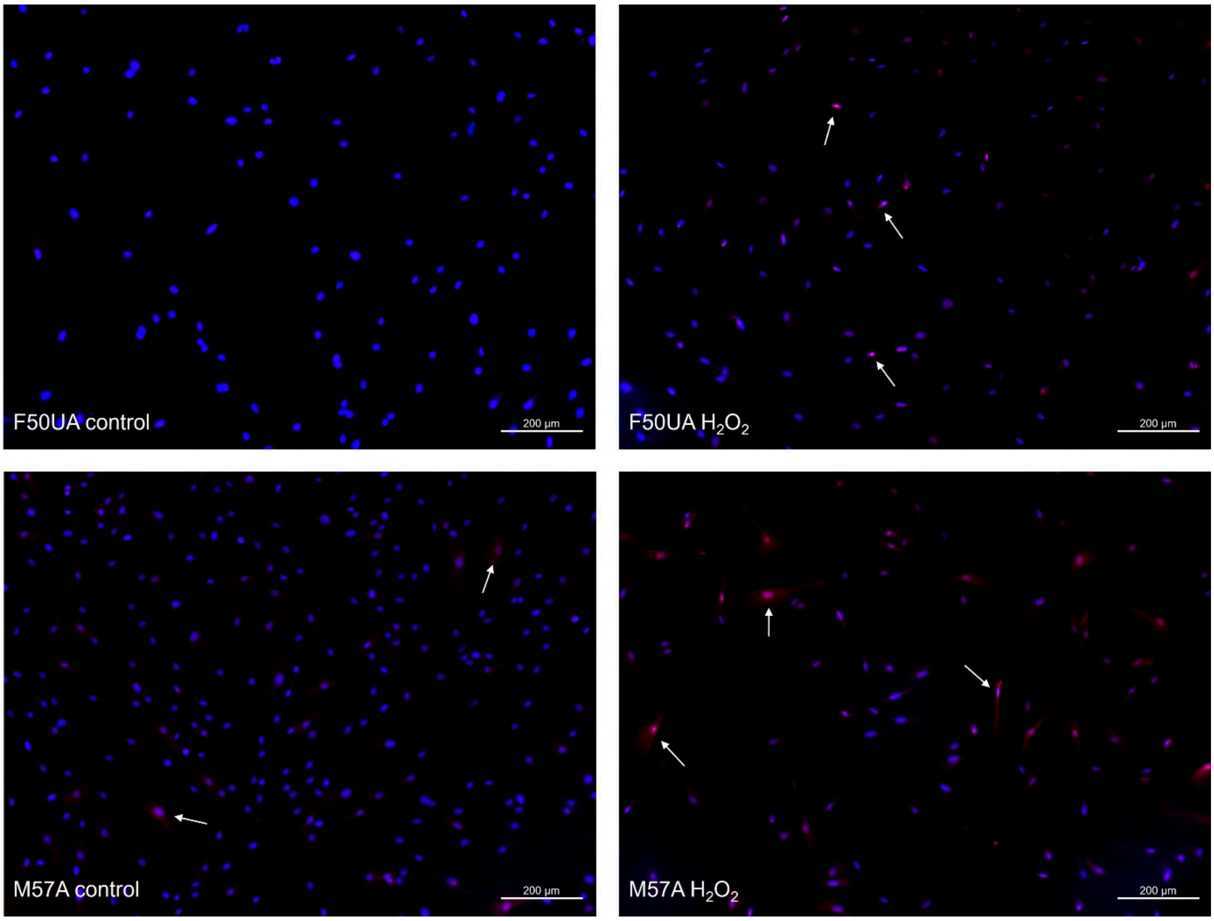

Figure S3. Representative overview images of p16 immunofluorescence staining in acute senescent proto-myofibroblasts. The white arrows in the figure mark exemplary p16 positive cells (red). The cell nuclei were counterstained with DAPI (blue). Three biological replicates per donor and condition were applied. The images were taken with a 100x magnification (scale bar: 200 µM).

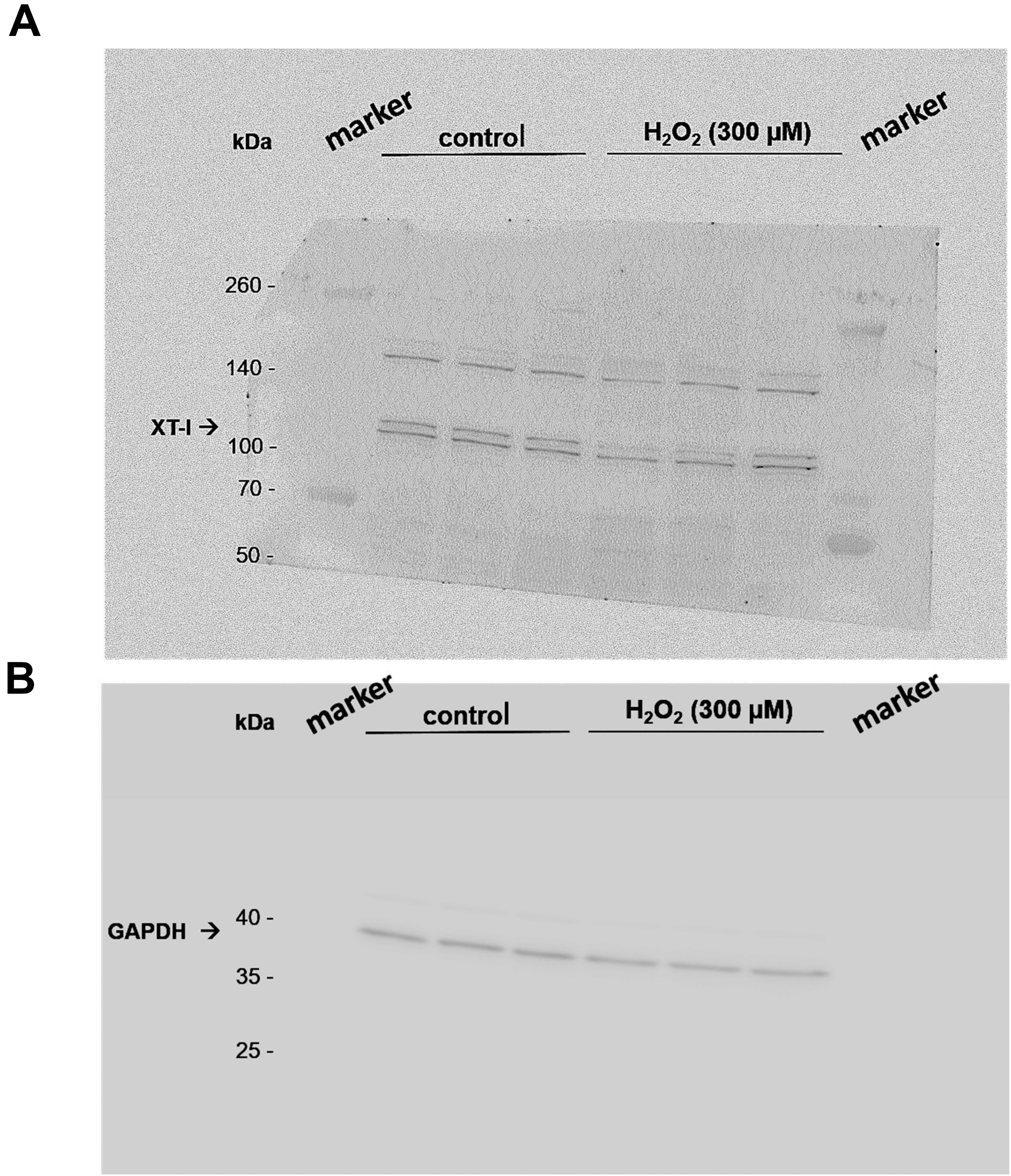

Figure S4. Analysis of XT-I protein expression in acute senescent proto-myofibroblasts. **(A)** Representative image of the XT-I protein detection by immunoblotting. Three biological replicates and one technical replicate per condition were applied (control and H_2O_2). **(B)** Detection of GAPDH protein expression was performed to normalize XT-I protein expression.

A

B

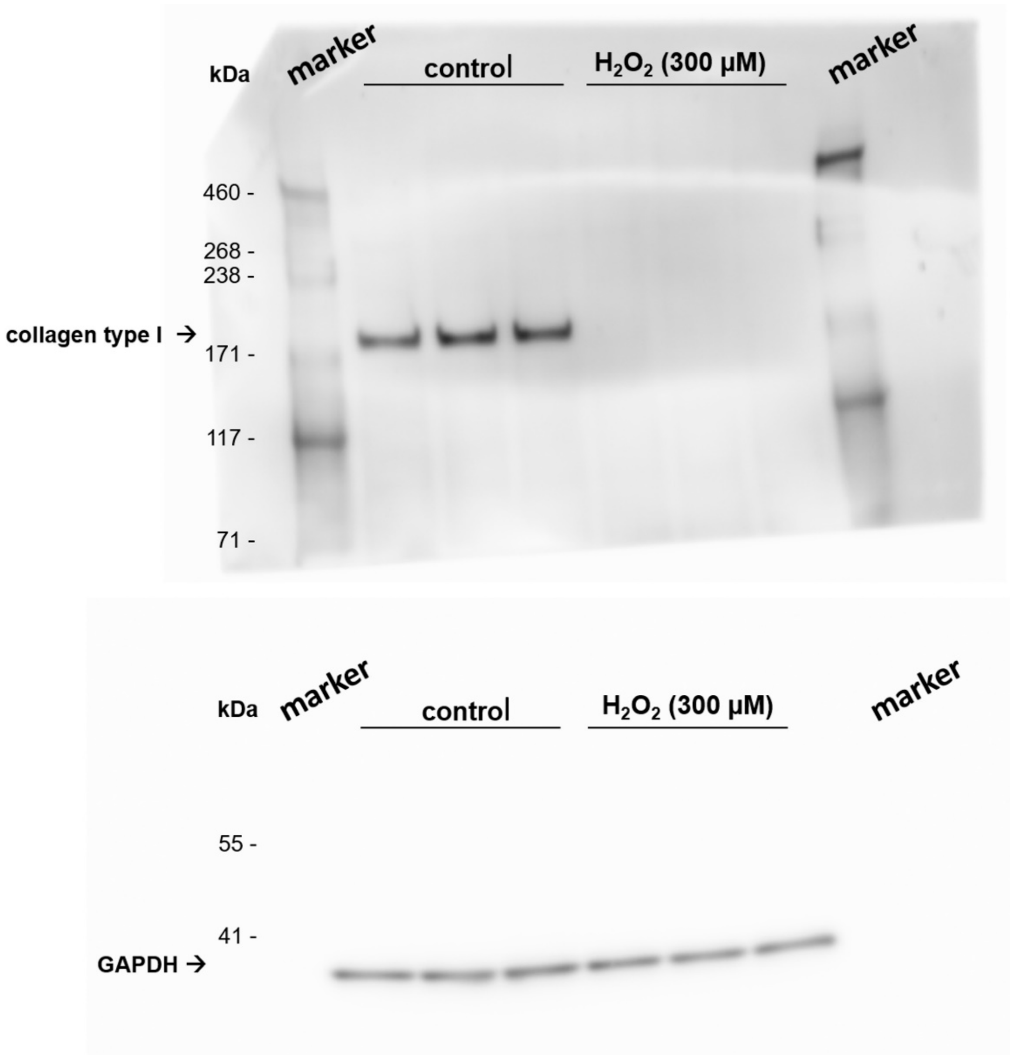

Figure S5. Analysis of collagen type I protein expression in acute senescent proto-myofibroblasts. **(A)** Representative image of the collagen type I detection by immunoblotting. Three biological replicates and one technical replicate per condition were applied (control and H_2O_2). **(B)** Detection of GAPDH protein expression was performed to normalize collagen type I protein expression.

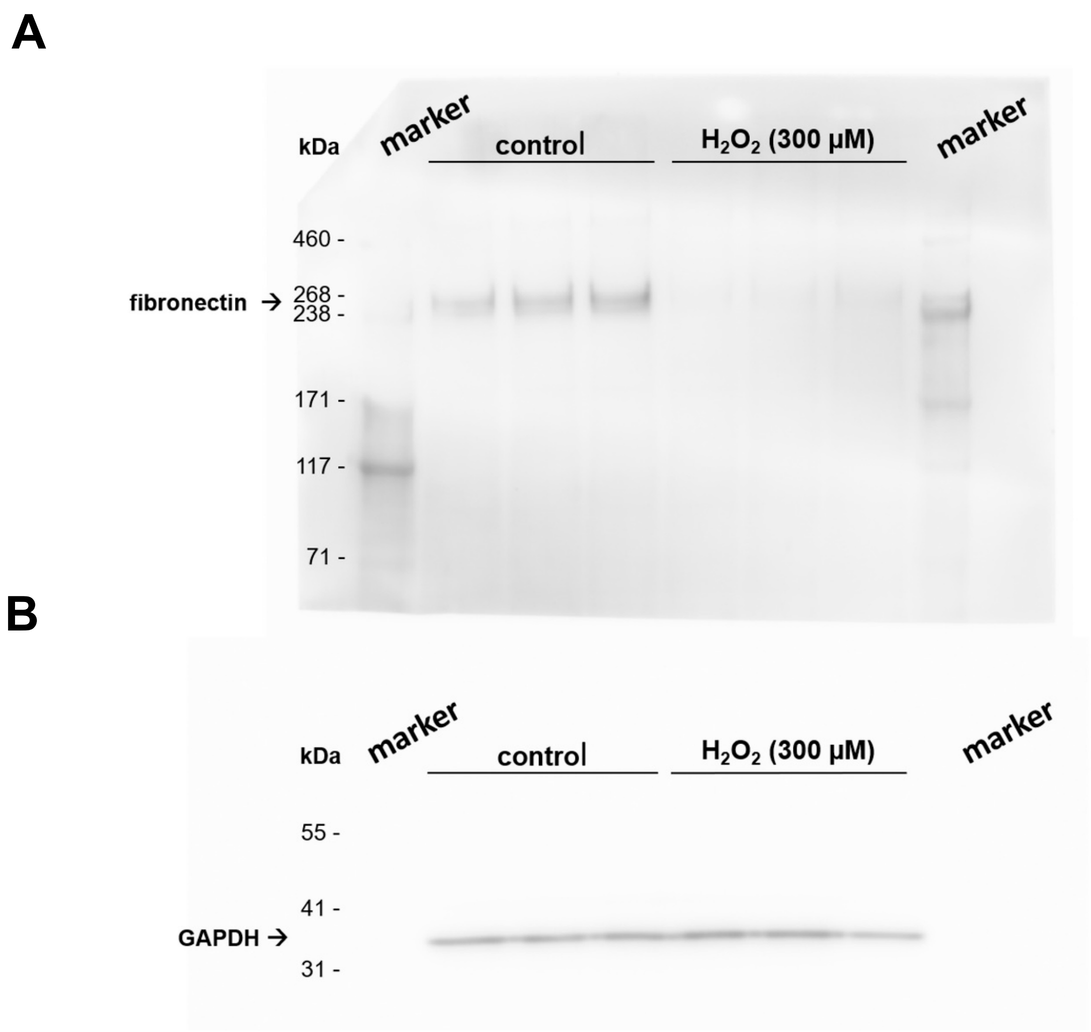

Figure S6. Analysis of fibronectin protein expression in acute senescent proto-myofibroblasts. **(A)** Representative image of the fibronectin protein detection by immunoblotting. Three biological replicates and one technical replicate per condition were applied (control and H_2O_2). **(B)** Detection of GAPDH protein expression was performed to normalize fibronectin protein expression.

A

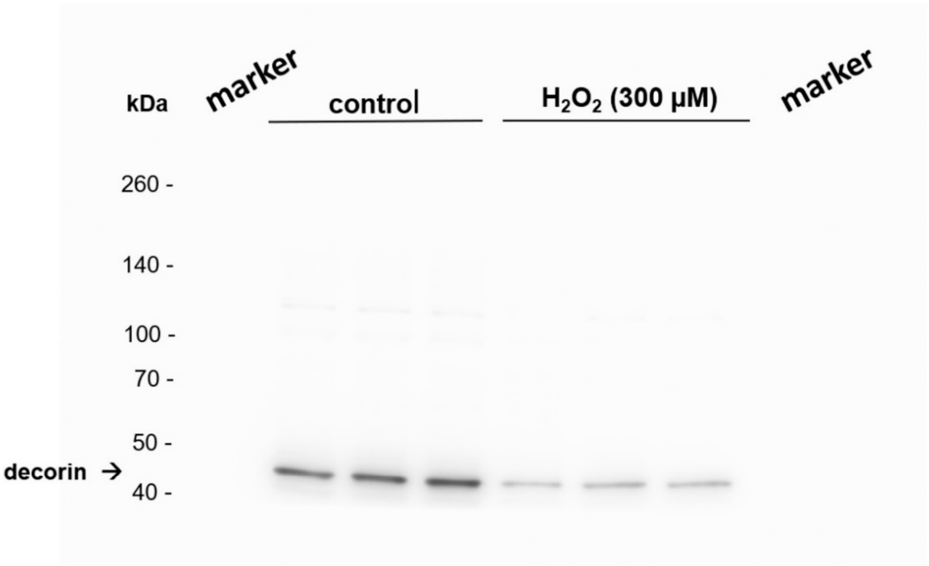

B

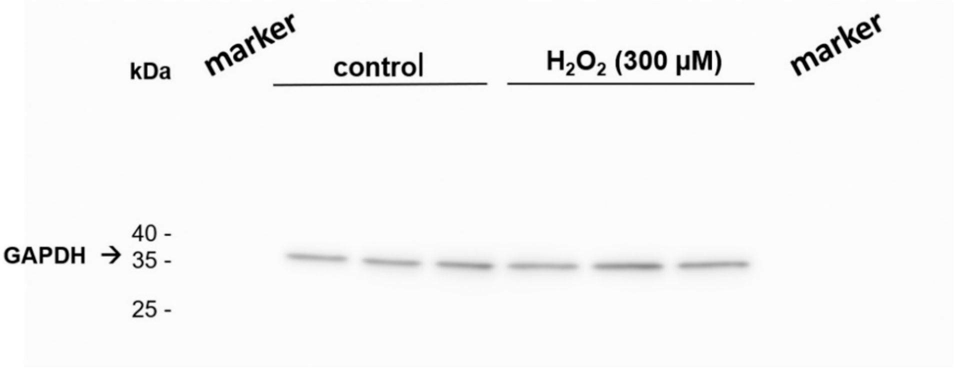

Figure S7. Analysis of decorin protein expression in acute senescent proto-myofibroblasts. **(A)** Representative image of the decorin protein detection by immunoblotting. Three biological replicates and one technical replicate per condition were applied (control and H_2O_2). **(B)** Detection of GAPDH protein expression was performed.to normalize decorin protein expression.
